# Food-grade TiO_2_ impairs intestinal and systemic immune homeostasis, initiates preneoplastic lesions and promotes aberrant crypt development in the rat colon

**DOI:** 10.1038/srep40373

**Published:** 2017-01-20

**Authors:** Sarah Bettini, Elisa Boutet-Robinet, Christel Cartier, Christine Coméra, Eric Gaultier, Jacques Dupuy, Nathalie Naud, Sylviane Taché, Patrick Grysan, Solenn Reguer, Nathalie Thieriet, Matthieu Réfrégiers, Dominique Thiaudière, Jean-Pierre Cravedi, Marie Carrière, Jean-Nicolas Audinot, Fabrice H. Pierre, Laurence Guzylack-Piriou, Eric Houdeau

**Affiliations:** 1 Toxalim (Research Centre in Food Toxicology), Université de Toulouse, INRA, ENVT, INP-Purpan, UPS, Toulouse, France; 2 Luxembourg Institute of Science and Technology (LIST), Materials Research and Technology (MRT), Advanced Instrumentation for Ion Nano-Analytics (IANA), L-4362 Esch-sur-Alzette, Luxembourg; 3 Synchrotron SOLEIL, F-91192 Gif-sur-Yvette, France; 4 French Agency for Food, Environmental and Occupational Health and Safety (ANSES), F-94701 Maisons-Alfort, France; 5 Université Grenoble-Alpes, INAC-LCIB, Laboratoire Lésions des Acides Nucléiques, 17 rue des Martyrs, F-38000 Grenoble, France; 6 CEA, INAC-SCIB, Laboratoire Lésions des Acides Nucléiques, 17 rue des Martyrs, F-38000 Grenoble, France

## Abstract

Food-grade titanium dioxide (TiO_2_) containing a nanoscale particle fraction (TiO_2_-NPs) is approved as a white pigment (E171 in Europe) in common foodstuffs, including confectionary. There are growing concerns that daily oral TiO_2_-NP intake is associated with an increased risk of chronic intestinal inflammation and carcinogenesis. In rats orally exposed for one week to E171 at human relevant levels, titanium was detected in the immune cells of Peyer’s patches (PP) as observed with the TiO_2_-NP model NM-105. Dendritic cell frequency increased in PP regardless of the TiO_2_ treatment, while regulatory T cells involved in dampening inflammatory responses decreased with E171 only, an effect still observed after 100 days of treatment. In all TiO_2_-treated rats, stimulation of immune cells isolated from PP showed a decrease in Thelper (Th)-1 IFN-γ secretion, while splenic Th1/Th17 inflammatory responses sharply increased. E171 or NM-105 for one week did not initiate intestinal inflammation, while a 100-day E171 treatment promoted colon microinflammation and initiated preneoplastic lesions while also fostering the growth of aberrant crypt foci in a chemically induced carcinogenesis model. These data should be considered for risk assessments of the susceptibility to Th17-driven autoimmune diseases and to colorectal cancer in humans exposed to TiO_2_ from dietary sources.

Titanium dioxide (TiO_2_) is a naturally occurring metal oxide and is one of the five engineered nanomaterials most commonly used in daily consumer products, including food^1^. The TiO_2_ food additive, referred to as E171 in the European Union (EU), is commonly used as a whitening and brightening agent in confectionary (candies and chewing gum), white sauces and icing^1^,^2^,^3^. The Food and Drug Administration approved the use of food-grade TiO_2_ in 1966 with the stipulation that TiO_2_ levels must not exceed 1% of the food weight^4^. In Europe, the current EU Directive 94/36/EC authorizes the use of E171 in foodstuffs without establishing an acceptable daily intake level by the Joint FAO/WHO Expert Committee on Food Additives, based on TiO_2_ absorption considered to be very low^5^. Nevertheless, the common use of E171 leads to significant levels of daily dietary intake of nanoparticulate matter among humans^1^. Indeed, E171 batches show broad size distributions of TiO_2_ primary particles (diameters of 30 to 400 nm), with up to 36% of particles falling below 100 nm in one dimension, i.e., nanoparticles (TiO_2_-NPs)^1^,^2^,^3^. TiO_2_-NPs have been easily isolated from food products such as chewing gum^6^. Human exposure analyses on foods consumed among American and British populations report that children under the age of 10 present the highest exposure level compared to adults (1–3 *vs*. 0.2–1 mg TiO_2_/kg of body weight (BW)/day, respectively)^1^. However, the oral route for TiO_2_ remains poorly investigated among toxicological testing studies, in contrast to issues of dermal contact or inhalation, i.e., the main routes for occupational exposure^1^,^7^. In addition, studies on the gastrointestinal (GI) uptake and effects of TiO_2_ have been primarily conducted based on NP models such as P25 Aeroxide^®^, which is referenced in the nanoparticle repository of the Joint Research Centre (JRC) (Ispra, Italy) as NM-105; in contrast to E171, these NP models are strictly nanosized^8^,^9^,^10^,^11^,^12^. Although most studies agreed for limited intestinal absorption of TiO_2_ in rats and humans^13^,^14^,^15^, a facilitated passage of TiO_2_-NPs through microfold cells (M-cells) lining the Peyer’s patches (PP) has been demonstrated *in vitro* and *in vivo*^8^,^16^. In humans, TiO_2_ particles of dietary origin have been found in the PP of patients suffering from inflammatory bowel disease (IBD)^17^ including infants^18^, and potent inflammasome activation has been reported *in vitro* using TiO_2_-NPs^19^. These studies point to possible contributions to chronic inflammatory processes in the gut if TiO_2_ particles accumulate in the cells of the PP through chronic dietary exposure, and this remains to be explored *in vivo* with the E171 food additive at relevant exposure levels for humans. Furthermore, TiO_2_ has been classified by the International Agency for Research on Cancer (IARC) as a possible human carcinogen in Group 2B after inhalation^20^ on the basis that inhaled or intra-tracheally administered nano- and fine-sized TiO_2_ induces lung cancer in rats^21^. Given the increasing number of commercial foods containing the TiO_2_ additive, *in vivo* experiments are required to determine whether chronic exposure to food-grade TiO_2_ particles may present risks of IBD and/or carcinogenesis in the exposed gut on a daily basis. In the present study, we examine the tissue distribution and immunotoxicity of E171 food-grade TiO_2_ orally administered over 7 days to rats at 10 mg/kg of BW/day in comparison to the NM-105 (i.e., P25) referent OECD nanomaterial. The patterns of intestinal inflammation, preneoplastic lesion development and colonic aberrant crypt foci (ACF) promotion were assessed in rats with or without dimethylhydrazine (DMH)-induced carcinogenesis following oral E171 treatment at the same dosage delivered over 100 days.

## Results

### Food-grade TiO_2_ particles cross the gut barrier and reach the liver without altering intestinal permeability or causing DNA damage in Peyer’s patches

**Figure 1:**
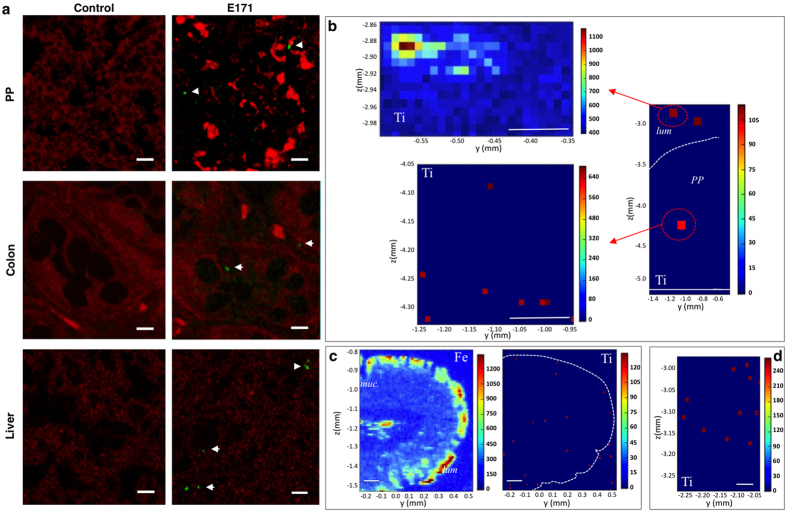
Tissue distribution of E171 particles in the rat intestine and liver after 7 days of oral exposure. (**a**) Confocal images of PP, colon and liver tissue sections from control and E171-treated rats showing tissue autofluorescence in red and light-scattering TiO_2_ particles in green (arrowheads) (scale bars 10 μm). (**b**–**d**) μXRF mapping of Ti distribution (red pixels) in PP (**b**), colon (**c**), and liver (**d**) tissue sections. In (**b**), the two left panels show Ti distribution in PP *vs*. the luminal side (lum) in higher resolution maps (10X) (scale bars 100 μm). In (**c**), note the presence of Ti overlaying iron (Fe)-rich epithelial cells lining the colonic mucosa (dashed line in the right panel) and Ti distribution in the mucosa (muc) (scale bars 100 μm). In (**d**), a tissue section from the liver in a Ti-rich area close to the portal vein sinus is shown (scale bar 50 μm).

Analysis of the particle size and crystal form of food-grade TiO_2_ shows that our E171 batch is a representative commercially sourced TiO_2_ food additive for our oral toxicity study (Supplementary Fig. S1 and SI section). Confocal and fluorescence reflection microscopy methods were used to examine the fate of TiO_2_ along the gut-liver axis in rats that were orally given ultrasonicated E171 particles in water. We first studied the dispersion state of TiO_2_ particles recovered from the luminal content of the jejunum and colon 4 h after a single dose of E171 was delivered. In comparison to the initial bolus, TiO_2_ particles did not reagglomerate *in vivo* when transiting along the gut (Supplementary Fig. S2). Upon absorption, light-diffracting TiO_2_ particles were found in the PP along the small intestine as well as in the colonic mucosa and liver of rats orally given E171 for 7 days but not in the controls (Fig. 1a and complementary TEM images in Supplementary Fig. S3). In the same rats, to ensure that the light scattering particles are primarily TiO_2_, we used μXRF for Ti element detection. As expected, Ti was detected in the gut lumen (i.e., corresponding to the residual bolus of E171 given to the rats) and PP (Fig. 1b) as well as in colon mucosa (Fig. 1c). In addition, Ti was found in the liver, with the highest density found close to the portal vein sinus, which collects blood from the intestine (Fig. 1d). Finally, we assessed whether oral exposure to E171 affected gut permeability *in vivo*, thereby facilitating particle absorption as a result of barrier disruption. No significant change in epithelial paracellular permeability to ^51^Cr-EDTA that was orally given to rats was observed in the E171 group in comparison to the controls (1.41 ± 0.08 *vs*. 1.63 ± 0.09% of total radioactivity recovered in 24 h urine samples, respectively; *P* = 0.1).

**Figure 2:**
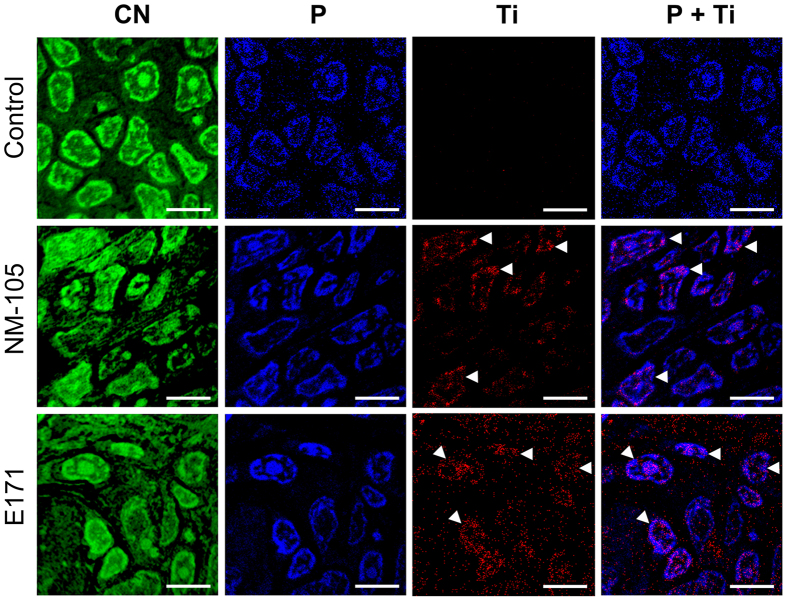
NanoSIMS analyses of subcellular Ti distribution in PP after 7 days of oral exposure to NM-105 or E171. The raster size was set to 20 × 20 μm^2^. NanoSIMS images for the elemental distributions of carbon-nitrogen ^12^C^14^N (green), phosphorus ^31^P (blue), and titanium oxide ^48^Ti^16^O (red) and the merged image of ^31^P and ^48^Ti^16^O (blue/red) on ultra-thin sections of PP (scale bars, 5 μm). The image overlay (P + Ti) shows Ti-rich zones in the nuclei of cells in PP (arrowheads).

To compare the subcellular distribution of Ti elements in rats orally given E171 or TiO_2_-NP model NM-105, we executed nanoscale secondary ion mass spectrometry (nanoSIMS) imaging with a beam size of 80–100 nm, allowing for the high-resolution mapping of the distribution of TiO clusters^22^ in rats orally dosed for 7 days. No Ti signal was detected in PP tissue sections of the control rats (Fig. 2), while Ti was found in the PP of all TiO_2_-treated rats, with similar distribution patterns between the NM-105 and E171 TiO_2_ sources (Fig. 2). The highest Ti density was found in the central zones of the PP, which are rich in immune cells (Supplementary Fig. S4a,b). In addition to Ti in the cytoplasm, Ti-rich regions were identified in the nuclei of PP cells and were closely associated with the phosphorus-positive chromatin (Fig. 2 and Supplementary Fig. S4b). Due to the nuclear translocation of Ti, the genotoxicity of both TiO_2_ compounds was evaluated (see SI section). No increase in DNA damage was detected in PP cells of the E171- and NM-105-treated rats (Supplementary Fig. S4c,d).

### Food-grade TiO_2_ particles affect dendritic cell frequencies and T cell populations in the Peyer’s patches and cause imbalances in intestinal and systemic immune responses

**Figure 3:**
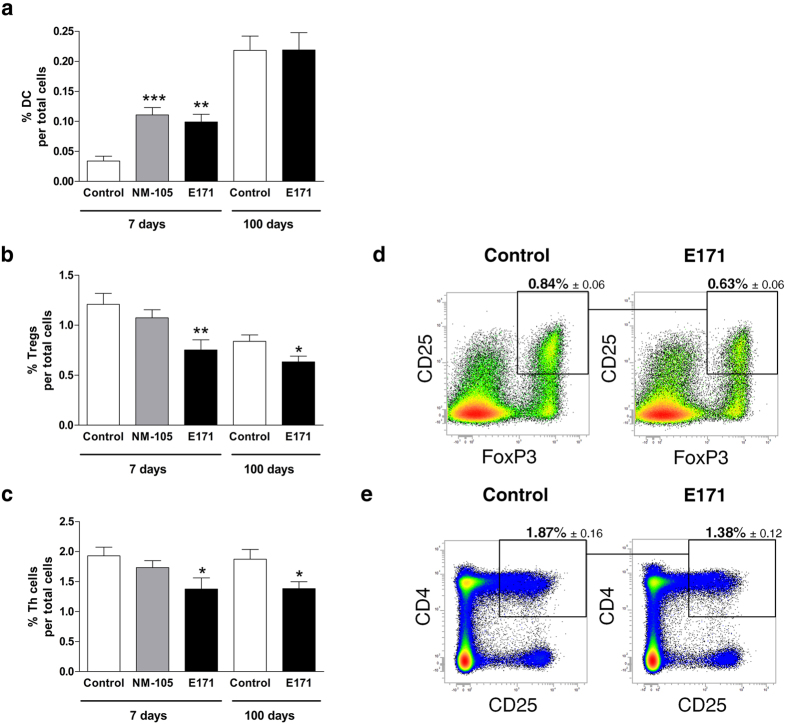
Frequency of dendritic, regulatory T and Th cells in Peyer’s patches following oral TiO_2_. Rats were orally exposed to 10 mg/kg of BW/day with NM-105 (grey bars), E171 (black bars) or vehicle (white bars) for 7 days by daily gastric gavage or for 100 days through the drinking water. The average frequency of DC cells (**a**), Tregs (**b**), and Th cells (**c**) in PP (n = 10 to 11 rats/group); representative effects of chronic E171 treatment for 100 days on Treg and Th cell populations based on FoxP3 and CD25 expression by CD4^+^ T cells (**d**) and CD25 expression in CD4+ T cells (**e**). All data are expressed as proportions and are written as the mean ± s.e.m. *P < 0.05, **P < 0.01, ***P < 0.001 *vs*. the control: one-way ANOVA followed by Tukey’s multiple comparison test for the 7-day treatment and a Student’s *t*-test for the 100-day treatment.

Resident dendritic cells (DC) in gut sample antigens from the lumen, and have important implications for tolerance and immune defences^23^. We first evaluated frequency of DC in the TiO_2_-treated rats, namely the CD11b/c^+^ CD103^+^ MHC-II^+^ DC, which are pivotal for immune tolerance as they induce regulatory T cells (Tregs)^24^,^25^. After 7 days of oral exposure, both NM-105 and E171 induced a significant increase in DC frequency in PP (Fig. 3a) without affecting the spleen at the systemic level (not shown). After chronic E171 treatment, early effects on DC in the PP were found to be transient, as they were not detected in rats exposed for 100 days through drinking water (Fig. 3a).

Regarding Tregs, the NM-105 nanomaterial had no effect on PP after 7 days of oral exposure (Fig. 3b) while the same duration of treatment with the E171 additive led to a significant decrease in this cell subset (i.e., CD4^+^CD25^+^FoxP3^+^) that was still observed in PP after 100 days of exposure (Fig. 3b,d). Interestingly, we found that decreased levels of Tregs appeared concomitantly with a decrease in CD4^+^CD25^+^ T helper (Th) cells, indicating failure of Th cell expansion (Fig. 3c,e). To determine whether TiO_2_ particles directly mediated T cell depletion, cells isolated from PP of untreated rats were exposed *ex vivo* to E171 particles or NM-105 TiO_2_-NPs, and cell viability and proliferation were compared. A dose-dependent cytotoxic and anti-proliferative effect on the T cells was observed, and this effect was found to be more pronounced with E171 compared to the NM-105 TiO_2_-NP model (Supplementary Fig. S5).

**Figure 4:**
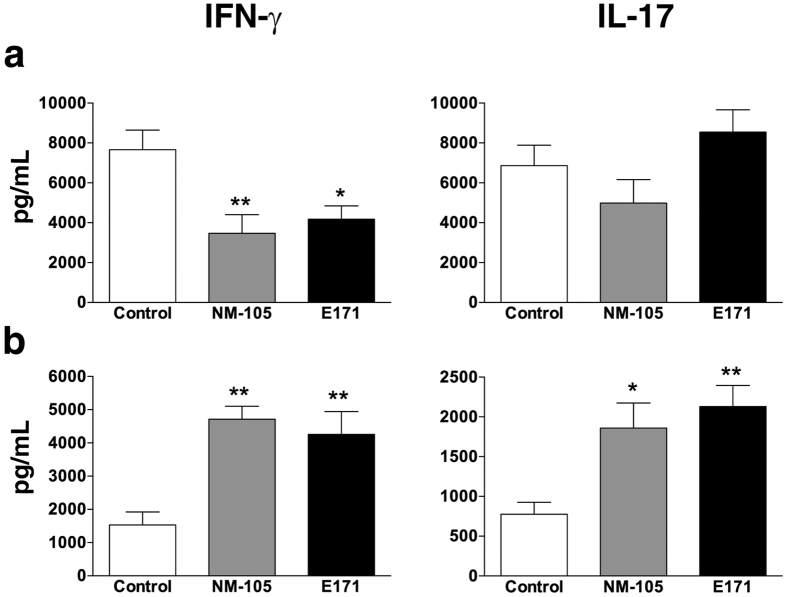
Oral TiO_2_ impairs CD3/CD28-induced T cell cytokine responses by isolated cells from Peyer’s patches and the spleen. Rats were treated *per os* with 10 mg/kg of BW/day of NM-105 (grey bars), E171 (black bars) or vehicle (white bars) over 7 consecutive days. Immune cells were harvested from PP (**a**) and the spleen (**b**) and restimulated *in vitro* with anti-CD3/CD28 antibodies for 4 days to induce cytokine secretion by T cells. Cytokine concentrations were evaluated in the cell supernatant *via* ELISA. The data are written as the mean ± s.e.m. (n = 10 rats/group). *P < 0.05, **P < 0.01 *vs*. controls. Statistical analyses were performed with one-way ANOVA followed by Tukey’s multiple comparison tests.

We then compared the effects of orally administering NM-105 and E171 particles to rats for 7 days on mucosal inflammation and immune cell responses in PP and the spleen. We did not detect any change in myeloperoxidase (MPO) activity, a marker of neutrophil infiltration, or in the content of basal cytokines (i.e., tumour necrosis factor (TNF)-α interleukin (IL)-10, IL-1β, interferon (IFN)-γ and IL-17) in mucosa of the small and large intestine relative to the control rats (Supplementary Table S1). To study *ex vivo* immune cell responses, total immune cells were isolated from PP and the spleen and then cultured with anti-CD3/CD28 antibodies to induce cytokine secretion into the culture media. In the PP, all TiO_2_ materials attenuated inflammatory IFN-γ secretion relative to the controls while the IL-17 response remained unchanged (Fig. 4a). In the spleen, both NM-105 and E171 elicited a potent Th1/Th17 immune response through increased production of IFN-γ and IL-17 (Fig. 4b).

### Food-grade TiO_2_ particles initiate and promote preneoplastic lesion formation in the colon and induce mucosal low-grade inflammation

**Figure 5:**
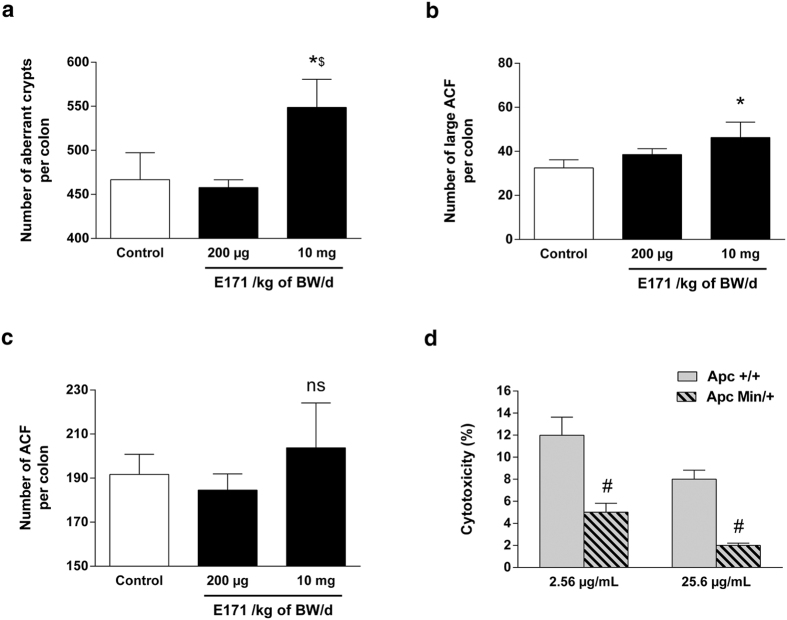
Exposure to food-grade TiO_2_ promotes colon carcinogenesis *in vivo* and induces preferential cytotoxicity in normal colon epithelial (Apc^+/+^) cells *in vitro*. After induction of cancer by DMH, rats were orally exposed to 200 μg or 10 mg/kg of BW/day of E171 in drinking water (black bars, 12 rats per group) or to water only (white bars, 12 rats) for 100 days: (**a**) The number of aberrant crypts per colon (AC/colon), (**b**) the number of large aberrant crypt foci per colon (large ACF/colon), and (**c**) the number of aberrant crypt foci per colon (ACF/colon). (**d**) Cytotoxic effects of 24 h of exposure to 2.56 μg/mL or 25.6 μg/mL of food-grade (E171) TiO_2_ on normal Apc^+/+^ and preneoplastic Apc^Min/+^ colon epithelial cells. The data are written as the mean ± s.e.m. (n = 6 for *in vitro* study and n = 12 rats/group for *in vivo* study), *P < 0.05 *vs*. control and ^$^P < 0.05 *vs*. 200 μg/kg of BW/day, one-way ANOVA followed by Tukey’s multiple comparison test; ^#^P < 0.05 *vs*. Apc^+/+^, Student’s *t*-test.

We first explored the promotion of preneoplastic lesions (i.e., ACF) *in vivo* in rats treated with DMH to initiate colon carcinogenesis. Rats were exposed to food-grade TiO_2_ in drinking water at 200 μg and 10 mg/kg of BW/day for 100 days, i.e., at doses approximating human dietary levels for adults and children^1^. The number and size of ACF (i.e., the number of lesions and the number of aberrant crypts per lesion) and the number of total aberrant crypts per colon were examined in a double-blind study. E171 treatment at 10 mg/kg of BW/day significantly increased the total number of aberrant crypts per colon as well as the number of large ACF per colon (i.e., more than three aberrant crypts per ACF) (Fig. 5a,b) relative to the control and 200 μg/kg of BW/day groups. Despite an increasing trend at the highest dose, no significant difference in the number of ACF per colon was observed between the groups of rats (Fig. 5c). To explain the growth-promoting effects on colonic preneoplastic lesions, we tested whether E171 differentially affects the viability of normal or preneoplastic cells through the comparative cytotoxicity of food-grade TiO_2_ particles on nonmutated (Apc+/+) cells and genetically defined preneoplastic (Apc Min/+) cells using an MTT assay. At the two concentrations tested, we found that 24 h exposure to E171 was more cytotoxic to Apc+/+ than to Apc Min/+ cells (Fig. 5d), hence providing an *in vitro* rationale for the selection of preneoplastic cells in early stages of carcinogenesis.

**Figure 6:**
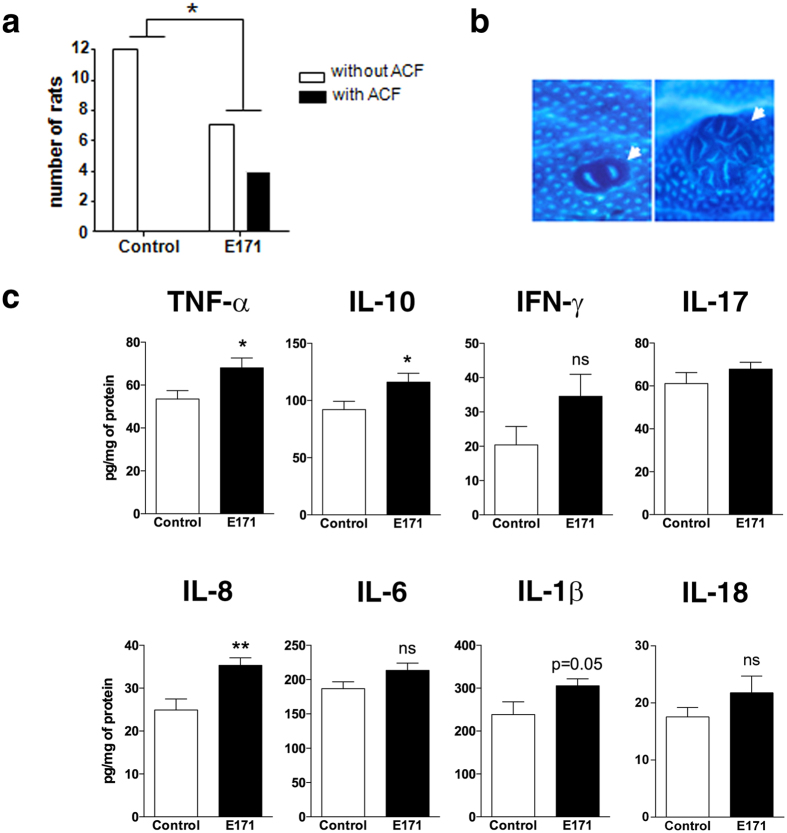
Chronic exposure to food-grade TiO_2_ triggers low-grade inflammation and initiates preneoplastic lesions. Rats were orally exposed to 10 mg/kg of BW/day of E171 in drinking water (black bars, 11 rats) or to water only (white bars, 12 rats) for 100 days. (**a**) The number of rats with or without aberrant crypt foci (ACF) in the colon and (**b**) ACF (arrowheads) at the colonic mucosal surface stained with methylene blue (40X) drawn from two ACF-positive rats. (**c**) Cytokine assays in colonic mucosa. The data are written as the mean ± s.e.m. (n = 10 to 11 rats/group). *P < 0.05, **P < 0.01 *vs*. the control: (**a**) contingency analysis followed by Fisher’s exact test for ACF analysis, and (**c**) Student’s *t* test.

We also determined whether chronic E171 treatment at 10 mg/kg of BW/day may initiate the spontaneous development of ACF in normal rats, i.e., without the induction of carcinogenesis by DMH. No ACF were observed in the colons of the control rats (Fig. 6a). Conversely, in the E171 group, 4 of the 11 animals spontaneously developed one to three ACF per colon (Fig. 6a). Three of the 4 rats developed lesions of 1 to 3 aberrant crypt(s) per ACF, and 1 rat developed a severe lesion of 12 aberrant crypts (Fig. 6b). Interestingly, cytokine assays showed moderate but significant increases in TNF-α (+26%, P < 0.05), IL-8 (+45%, P < 0.01), and IL-10 (+26%, P < 0.05) in the colonic mucosa of E171-treated rats relative to the controls (Fig. 6c). Western blotting for caspase-1 did not show cleaved caspase-1 in the colons of E171-treated rats relative to control animals (Fig. S6), indicating the absence of inflammasome activation into the mucosa; accordingly, no significant change in the downstream caspase-1 effectors IL-1β and IL-18 (Fig. 6c) were found in our experimental setting using a low dose of E171.

## Discussion

Titanium dioxide, which is manufactured as a food ingredient (and referred to as E171), is ingested daily as mixed nano- and submicron-sized particles in the human diet^1^. While recent reports based on NP models show that TiO_2_-NPs translocate through the intestinal epithelia, no *in vivo* study has been carried out to investigate the tissue distribution of food-grade TiO_2_ particles along the gut and whether the nanoscale fraction of E171 particles presents a specific risk *via* the oral route. Our study shows that ultrasonicated E171 particles prepared in water before oral administration to rats did not reagglomerate *in vivo* in the intestinal lumen. Transepithelial passage occurred in the jejunum and the colon after one week of treatment, and the titanium (Ti) reached the liver, exhibiting systemic absorption of E171 as previously reported based on TiO_2_-NP models^13^,^14^,^26^,^27^. This first indicates that the daily consumption of E171-containing food may constitute a persistent source for the systemic passage of TiO_2_-NPs and that particles sequestered into the gut mucosa represent an unexplored topic for *in vivo* toxicity assessments of food-grade TiO_2_. No change in intestinal permeability was observed, indicating that particle absorption did not result from a loss of epithelial barrier integrity after TiO_2_ treatment. The relatively low density of Ti signals in the liver suggested limited hepatic retention after one week of daily dosing. This is in accordance with previous oral studies using NP models, wherein low Ti levels (<0.03 to 0.2 μg Ti/g of tissue) were detected in the liver and were not found to accumulate after 5 days of daily oral exposure at a similar dose^14^ or after 13 weeks of treatment at higher doses (>250 mg/kg of BW/day)^13^.

In humans, TiO_2_ absorption into the bloodstream has been recently reported for healthy volunteers orally given a single dose of a pharmaceutical/food-grade TiO_2_ formulation^28^. In this pilot study, because lumen-to-blood TiO_2_ passage was found within 2 h after ingestion, the authors concluded that particle uptake is limited to the small intestine. Using rats exposed daily for one week, we provide evidence for the occurrence of TiO_2_ absorption not only in the small intestine but also in the colon. It is likely that the slow transit time in the large intestine is responsible for TiO_2_ accumulation in the colonic lumen after repeated oral intake, forming a reservoir that could favour local absorption by epithelial cells. Importantly, the colon epithelium in rats and humans is a region rich in mucus-producing goblet cells, and a recent study using human Caco-2/HT29-MTX cell co-culture (i.e., a model of goblet cells) clearly showed that TiO_2_-NPs are preferentially entrapped by cells in the HT29-MTX co-culture model compared to Caco-2 cells cultured alone as regular enterocytes^8^.

In the human-focused study using a single dose^28^, lumen-to-blood TiO_2_ translocation began early in the small bowel but peaked 6 h after ingestion. This delayed passage is thought to be a result of PP uptake due to an avid capture of TiO_2_ particles by antigen-presenting M-cells lining the dome of the PP. A facilitated translocation pathway for TiO_2_-NPs has been demonstrated *in vitro* using a cell model of follicle-associated epithelium mimicking M-cells^8^. In our *in vivo* study, μXRF and nanoSIMS clearly showed Ti internalization in PP cells of rats orally exposed to food-grade TiO_2_. Given that the high resolution of nanoSIMS images (i.e., 80–100 nm) allows subcellular cartography, it is noteworthy that E171 titanium reached not only the cytoplasm of PP cells but also the nucleus. Similar Ti internalization was observed with pure nanoparticulate TiO_2_ matter (i.e., NM-105), suggesting that the nanoscale particle fraction of TiO_2_ in the E171 additive also distributes to immune cells following uptake by PP. Using Raman imaging, high levels of nuclear TiO_2_-NP uptake have been recently reported *in vitro* in lung epithelial cells^29^. In the liver, *in vivo* studies have shown that anatase TiO_2_-NPs (i.e., the main crystal phase of our E171 sample) can covalently bind to DNA^30^,^31^. In addition, we cannot exclude the possibility of the partial solubilisation of Ti from E171 particles, thereby favouring the nuclear uptake of metal ions as reported *in vitro* after 7 days of contact with body fluids^32^. In our study, it is noteworthy that no DNA damage, either as DNA strand breaks or oxidative DNA damage, was detected in PP despite the nuclear translocation of Ti elements after 7 days of oral treatment, hence demonstrating the absence of genotoxicity *in vivo* at low doses. Numerous studies have evaluated the genotoxicity of TiO_2_^7^, mainly following pulmonary, dermal or intravenous exposure, and discrepancies exist regarding the potential for genotoxicity for TiO_2_-NPs *in vivo*^7^,^33^,^34^. Importantly, TiO_2_-NPs have been recently reported to be non-genotoxic in the peripheral blood and the liver in mice exposed intravenously to 2, 5 or 50 mg/kg of BW per week for 4 consecutive weeks, as shown using various sensitive methods, including alkaline comet assays such as those used herein^34^.

Regarding immune regulatory effects, we show that TiO_2_ absorption (either with NM-105 or E171) led to an imbalance in resident antigen-presenting DC populations, promoting their accumulation in PP but having no effect on splenic DC, i.e., at the systemic level. Nevertheless, the accumulation of DC at PP was not observed in the 100-day study of E171, suggesting the existence of compensatory mechanisms after long-term exposure that normalize DC domiciliation in the gut. In contrast, a decreased frequency of immunoregulatory Tregs was observed in the PP of E171-treated rats only, an effect still observed after 100 days of oral treatment. This indicates that chronic exposure to food-grade TiO_2_ limits the expansion of intestinal Tregs, which have immunosuppressive properties that play a crucial role in the induction of oral tolerance and in the prevention of food allergy development while dampening proinflammatory responses in the gut^23^. Interestingly, we report that the E171-induced Treg defect in PP occurred concomitantly with a reduction in Th cells, suggesting that defects in Th cell differentiation dampen the expansion of all Th subsets, including Tregs. Consistently, our *in vitro* data show dose-dependent cytotoxic and anti-proliferative effects of TiO_2_ on T cells isolated from PP. Of note, this effect was found to be more pronounced for E171 particles than for the NM-105 NP model, and this may explain the decreased expansion of Th cells observed *in vivo* following E171 treatment. Finally, the absence (*in vivo*) or limited (*in vitro*) impacts of the TiO_2_-NP model with NM-105 on Treg and Th cell subsets suggest that the TiO_2_-induced reduction in T cell expansion can be mostly attributed to the larger particles that predominate in the E171 food additive.

After passage in the bloodstream, we found Th1/Th17 immune deviation in the spleen regardless of the TiO_2_ treatment group, demonstrating imbalanced immune responses at the systemic level. Titanium accumulation has been recently reported in the spleen after short-term oral exposure (i.e., 5 days) to low doses of TiO_2_-NP in rats^27^. Based on a comparable dosage and exposure duration, our data indicate that the systemic absorption of food-grade TiO_2_ particles could trigger an inflammatory profile in splenocytes, mainly through excessive Th17 responses without changes in total splenic T cell populations. Importantly, high IL-17 production has reportedly been associated with the pathogenesis of autoimmune diseases such as multiple sclerosis and rheumatoid arthritis^35^,^36^.

In the intestinal tract, it has been suggested that TiO_2_ particles may cause mucosal inflammation when they are taken up by immune cells^19^,^37^. Clinical studies have also highlighted the possibility that the accumulation of fine and ultra-fine dietary TiO_2_ particles in human PP could participate in the aetiology of IBD e.g., Crohn’s disease (CD)^17^,^18^. However, although Tregs are critical for preventing intestinal inflammation, paradoxically, a high level of Tregs has been found in the mucosa of IBD patients^38^. Consequently, the current evidence that chronic E171 treatment decreases Treg frequency at PP sites does not present long-term oral exposure to food-grade TiO_2_ as an environmental factor favouring the high Treg frequency in IBD mucosa. In addition, inflammation and disease progression are characterized by neutrophilia and gut barrier disruption in response to the high tissue content of a wide variety of inflammatory mediators, such as Th1 (IFN-γ) and Th17 cytokines (IL-17), IL-1β, TNF-α, IL-6, IL-8 and IL-10^39^. After one week of oral treatment with NM-105 or E171, we observed no change in intestinal permeability levels, no significant differences in neutrophil infiltration (assessed based on tissue MPO enzyme activity) and no significant differences in mucosal cytokine content along the gut. Another oral study in mice reported increased Th1 cytokine levels in the small intestine after a 10-day exposure to TiO_2_-NPs but this study used a 10-fold higher dosage^40^. Furthermore, while IFN-γ is an important contributor to IBD development^41^, here, we report a marked drop in its secretion levels when PP cells isolated from E171- or NM-105-treated rats were challenged with CD3/CD28 antibodies. This result contrasts with the behaviour of T cells isolated from IBD patients, which present high IFN-γ secretion levels when restimulated *in vitro*^42^. Our study indicates an immunosuppressive effect of TiO_2_ at initiator sites of proinflammatory processes that may be linked to TiO_2_-induced depletion of Th cells, as reported herein. However, our 100-day study shows that chronic exposure to a low dose of E171 moderately increases the mucosal content of IL-6, TNF-α, IL-8 and IL-10 in the rat colon without triggering significant changes in other inflammatory cytokines such as IFN-γ, which normally flares in rodent models of acute colitis^43^,^44^ and in CD patients^41^,^45^. The low magnitude of local cytokine production compared to that of untreated rats confirms that long-term oral exposure to food-grade TiO_2_ does not trigger IBD-like colitis, but rather, favours the development of low-grade inflammation in the colon. In previous *in vitro* studies, TiO_2_ microparticles have been reported to exhibit adjuvant activity in a pre-existing immune response^37^. More recently, the oral administration of pure TiO_2_-NPs at high doses (50 and 500 mg/kg of BW/day) to mice with dextran sulfate sodium (DSS)-induced colitis was found to worsen disease activity through the activation of the NLRP3 inflammasome^46^. The inflammasome is a central regulator of intestinal homeostasis and is a key factor affecting the development of intestinal inflammation through caspase-1 cleavage and the downstream production of IL-1β and IL-18^47^. From our long-term study using the E171 additive at a low dose, we report that food-grade TiO_2_ particles did not induce inflammasome activation in rats without previous defective intestinal barrier function. In line with this finding, we did not observe any change in IL-18 levels in the colonic mucosa. Only a small increase in IL-1β secretion at the limit of significance was observed, and hence without signs of a potent inflammatory cascade in the tissues. Therefore, the present study supports the hypothesis that translocation of TiO_2_ particulate matter in the colon following chronic exposure to E171 evokes a mild inflammatory response in the mucosa, while according to a recent study a greater deleterious effect involving inflammasome activation might occur when the gut is already faced with pathogenic challenges, such as a preexisting colitis^46^.

Importantly, our long-term study now highlights E171 as a risk factor in the promotion of preneoplastic lesions in the rat colon. In this study, which was performed using two doses representing human dietary levels, E171 treatment at 10 mg/kg of BW/day significantly increased the number of total aberrant crypts per colon as well as the number of large ACF per colon, showing a greater lesion area and increased severity of preneoplastic lesions, respectively. The consequent impact on the number of large ACF is noteworthy because ACF size more closely reflects the rate of tumour incidence than the quantity of ACF in rats^48^ as is the case for humans^49^. Very recently, Urrutia-Ortega *et al*.^50^ showed that the intragastric administration of E171 at 5 mg/kg of BW/day over 10 weeks exacerbated tumour formation in a chemical colitis-associated cancer (CAC) model, i.e., in mice with severe and chronic mucosal inflammation induced by multiple cycles of dextran sodium sulfate (DSS) treatment after azoxymethane cancer induction^51^. In contrast, the promotion of ACF by food-grade TiO_2_ particles was herein observed in normal mucosa, demonstrating that E171 can promote the development of preneoplastic lesions in rats without pre-existing epithelial barrier injuries. *In vitro*, we report that a single Apc mutation rendered intestinal epithelial cells resistant to TiO_2_-driven cytotoxicity relative to normal colonocytes. This suggests a survival advantage for premalignant cells when subjected to long-term TiO_2_ exposure, which may be a mechanism for ACF promotion *in vivo*. Because a similar *in vitro* effect was found for TiO_2_-NP model NM-105 (Supplementary Fig. S7), it is suggested that the promotional effect of E171 in our premalignant model may be linked to the nanosized fraction of TiO_2_ particles present in the food additive.

We further explored the effect of food-grade TiO_2_ particles on the initiation of carcinogenesis, i.e., in rats orally exposed to E171 for 100 days without cancer induction by DMH. Nearly 40% of the animals spontaneously developed preneoplastic lesions in the colon, suggesting that E171 can independently trigger the preneoplastic stages of carcinogenesis in the large intestine. Non-food-grade TiO_2_ pigment has already been classified by the IARC as potentially carcinogenic to humans (i.e., Group 2B) after inhalation^20^,^21^. In our oral study, we used the quantity, size and dysplastic features of colon ACF as established biomarkers of the adenoma-to-carcinoma sequence in rodents^48^ as in humans^52^. Among rats with spontaneous initiation, one animal displayed an ACF of 12 aberrant crypts, a lesion related to a microadenoma^53^. In addition, ACF initiation in E171-exposed rats correlated with the development of a microinflammatory environment in the colon as noted above. More studies must be conducted to identify mechanisms that initiate premalignant cells, but it is possible that the increased colonic content of TNF-α and IL-8, which is already known to play a central role in colorectal carcinogenesis^54^,^55^,^56^, may contribute to this pathogenic feature.

In summary, we report that the immunotoxicity of food-grade TiO_2_ (E171) particles after oral exposure in rats at low doses impairs intestinal immune homeostasis after one week of treatment. Th1/Th17 immune deviation in the spleen elicited by the E171 additive and the TiO_2_-NP model support proinflammatory potential at the systemic level for the nanosized TiO_2_ fraction present in the food additive. Furthermore, chronic exposure to E171 particles may initiate and promote the expansion of preneoplastic lesions in the colon, which parallels the development of an inflammatory microenvironment in the mucosa, and the selection of preneoplastic cells *in vitro*. Altogether, the current results emphasize that oral exposure to TiO_2_-based food additives should be investigated for human risk assessment as putative dietary factors contributing to Th17-driven autoimmune complications and to the development of colorectal cancer.

## Methods

### Particle preparation

The E171 sample was obtained from a French commercial supplier of food colouring. The referent P25 (NM-105) nanomaterial was provided by the European Union Joint Research Centre (EU JRC) as a test material of manufactured TiO_2_-NPs (P25 AEROXIDE^®^) and was selected by the Organization for Economic Cooperation and Development (OECD) for safety evaluations of titanium-based nanomaterials^57^. The TiO_2_ products were prepared following the generic Nanogenotox dispersion protocol^58^,^59^. Methods for the physicochemical characterization of the particles are presented in the SI section.

### Animals and experimental design

Adult male Wistar rats (175–200 g) were purchased from Janvier Labs (France). All animal experiments were performed in accordance with the guidelines of European legislation (Council Directive 2010/63/UE) and French Decree 2013-118 on the protection of animals used for scientific purposes and were approved by the Local Animal Care and Use Committee (TOXCOM-0036-EH-EH) of Toulouse Midi-Pyrénées (agreement CEEA-86). The animal facilities used are licensed by the relevant local authorities for rodents (agreement C31 555 13). In a first series of experiments, rats (n = 10 rats/group) were dosed daily by intragastric gavage (200 μL) with TiO_2_ NM-105, E171 (10 mg/kg of BW/day) or vehicle (water) for 7 days. Animals were used for tissue imaging, flow cytometry and cytokine assays and to carry out tissue inflammation and gut permeability measurements (additional methods are shown in the SI section). In a second series of experiments, groups of rats (n = 11 to 12 per group) were treated or not with 1,2-dimethylhydrazine (DMH) to induce colon carcinogenesis and were exposed to E171 at 200 μg or 10 mg/kg of BW/day through drinking water for 100 days. Control animals (n = 12) received water only. Rats were used for flow cytometry and cytokine assays and for gut inflammation and ACF assessments. In a third series of experiments, untreated rats (n = 4) were used for *ex vivo* cytotoxicity and proliferative assays on isolated immune cells. Finally, the E171 particle agglomeration state was followed using confocal microscopy in the luminal contents of the jejunum and colon collected from 4 rats 4 h after a single dose of 10 mg/kg was delivered.

### Confocal microscopy and micro X-ray fluorescence imaging

Tissue samples were fixed in 4% formaldehyde, equilibrated in 30% sucrose in phosphate buffer, frozen in liquid nitrogen and stored at −80 °C. Cryo-sections (15 μm thickness) were fixed in acetone at −20 °C (5 min), rehydrated in PBS (10 min), and then mounted in Pro-long gold antifade medium (Life Technologies). Tissue sections were examined under a confocal microscope (Leica SP8) at 488/BP 488–494 nm to detect light scattering TiO_2_ particles and at 514/BP 560–660 nm to monitor autofluorescence in the tissue. Similar measurements of E171-TiO_2_ light scattering at 488/BP 488–494 nm were performed for particle size determination in the E171 water suspensions given to the rats and in the recovered intestinal luminal contents (jejunum and colon) after their spreading, drying and mounting in Pro-long medium. The particles were detected using a 63X objective and a magnification factor of 1 pixel to 50 nm. The μXRF analyses were performed at the SOLEIL Synchrotron (DiffAbs beamline) using a 4-element silicon drift detector (Vortex-ME4, Hitachi). The Ti distribution in the tissue sections was determined using μXRF in micro-beam mode by adding secondary focusing optics. At the sample position, the beam size was 10.4 × 7.0 μm^2^ (horizontal and vertical, respectively) with a flux close to 10^10 ^phs^−1^. The energy of the monochromatic X-ray beam was set at 7.5 keV, and a 4-element silicon drift detector allowed for fluorescence collection.

### NanoSIMS imaging

The SIMS image analyses were performed on 200–300 nm ultra-thin sections prepared according to standard transmission electronic microscopy protocols and using a NanoSIMS50 (Cameca, France) equipped with a caesium source^22^. With an impact energy of 16 keV and a primary current of 1pA, the Cs^+^ beam was rastered over a 20 × 20 μm^2^ area on the sample surface. The images were recorded in 256 × 256 pixels with a counting time of 20 ms/pixel. The beam size was in the range of 80–100 nm^22^. The instrument was tuned for a mass resolution M/ΔM > 5000. The four masses recorded were the negative clusters ^12^C^14^N^−^ (*m*/*z* = 26.003 u), ^31^P^16^O^−^ (*m*/*z* = 46.969 u), ^46^Ti^16^O^−^ (*m*/*z* = 61.948 u) and ^48^Ti^16^O^−^ (*m*/*z* = 63.943 u). Due to his low affinity with one electron, the titanium (electron affinity Ea = 0.079 eV) was recorded as TiO clusters (Ea = 1.30 eV). Mass calibrations were achieved using standard references (Ti-sheet, Goodfellow). Both clusters of Ti isotopes (^46^Ti and ^48^Ti) were recorded simultaneously to verify the isotopic ratio (^46^Ti/^48^Ti = 0.112) and the separation of the isobaric masses (e.g., ^32^S^16^O_2_, and ^48^Ti^16^O).

### Intestinal permeability measurement

*In vivo* intestinal permeability was assessed using ^51^Cr-EDTA (Perkin Elmer Life Sciences), a marker of paracellular permeation. Briefly, rats were placed in metabolic cages for 3 days to become accustomed to the environment, and 0.7 μCi of ^51^Cr-EDTA diluted in 0.5 mL of saline was administered by gavage, and then urine samples were collected for 24 h. The total intestinal permeability to ^51^Cr-EDTA is expressed as a percentage of the administered radioactivity recovered from the 24 h urine samples as measured by a gamma counter (Cobra II, Packard).

### Cell isolation and flow cytometry analysis

Jejunal and ileal PP were washed 5 times with PBS 3 mM EDTA, once with PBS, and digested with collagenase for 40 min. Cell suspensions were filtered and purified by centrifugation on a 40–80% Percoll gradient. Spleen cells were filtered (40-μm nylon mesh filter) in PBS containing 1% FCS. Cell phenotypes were analysed with antibodies directed against CD103 (OX-62, Biolegend), MHC-II (OX-6, Biolegend) and CD11b/c (OX-42, BD Pharmingen) to determine DC phenotypes; CD4 (W3/25, BD Pharmingen) and CD25 (OX-39, BioLegend) were used to identify Th cells, and association with intracellular staining for FoxP3 (FJK-16s, eBioscience) was used to detect Tregs. The data were collected using a MACSQuant analyser (Miltenyi Biotec) and were analysed *via* VenturiOne software (AppliedCytometry).

### Cell culture and induction of cytokine response

Isolated cells from PP and spleen were seeded on 24-well plates coated with anti-CD3/CD28 antibodies (G4.18 and JJ319 eBioscience, respectively) at 1 * 10^6 ^cells/well in Cerottini medium^60^. After 4 days of antibody stimulation, the culture supernatants were collected and frozen at −80 °C until cytokine assays were performed.

### Cytokine assays

The levels of IL-6, IFN-γ, TNF-α, IL-1β, IL-10, IL-8 (CINC-1), and IL-17 in cell culture supernatants and/or tissue extracts were determined using commercial ELISA kits (Duoset R&D Systems) according to the manufacturer’s instructions. For tissue samples, segments of jejunum and colon were prepared in RIPA buffer (0.5% deoxycholate, 0.1% SDS, and 1% Igepal in TBS) containing complete protease inhibitor cocktail (Roche), and protein concentrations were measured using a BCA Optima kit (Interchim). The data are expressed as picograms per millilitre of cell culture medium supernatant or as picograms per milligram of tissue protein.

### Colon cell lines and cytotoxic assay

Apc +/+ (normal) and Apc Min/+ (preneoplastic, mutated on the *Adenomatous polyposis coli* gene) colon epithelial cells were established as previously described^61^. These cells harbour a temperature-sensitive mutation of the simian virus 40 large tumour antigen gene (tsA58) under the control of IFN-γ. All of the cells of these mice are ‘immortalized’, and they express active SV40 at the permissive temperature (33 °C). The cells were cultured at the permissive temperature of 33 °C in Dulbecco’s modified Eagle’s medium (DMEM) supplemented with 10% foetal calf serum, 2% penicillin/streptomycin, 2% glutamine and 10 U/mL IFN-γ. The experiments were performed at the non-permissive temperature of 37 °C and without IFN-γ to inhibit the SV40 transgene and to limit proliferation. At non-permissive temperatures, cell lines can be maintained in culture for 8 days, which is comparable to that of normal epithelial cells.

For the cytotoxicity assay, cells used were seeded into 96-well culture plates at a seeding density of 3.2 × 10^4^ cells per well in DMEM. The cells were cultured at 33 °C under permissive conditions until subconfluence occurred, and then they were transferred to non-permissive conditions for 24 h. After 24 h, the cells reached confluence, and the culture medium was replaced with TiO_2_ suspension in DMEM (2.56 μg/mL or 25.6 μg/mL). After TiO_2_ was added, the cells were incubated at 37 °C for another 24 h. The cells were then washed, 100 μl of (3-(4,5-dimethylthiazol-2-yl)-2,5-diphenyltetrazolium bromide tetrazolium (MTT) solution [0.45 mg/mL in phosphate-buffered saline (PBS)] was added to each well, and the cells were incubated at 37 °C for another 4 h. The reaction product was solubilized in 100 μl of lysis buffer (10% SDS, 0.1 M NaOH) before the colour of the reaction product was quantified using a plate reader at 570 nm.

### Models of aberrant crypt foci initiation and promotion

In a first series of experiments, to examine the impact of E171 on the promotion of ACF, after 5 days of acclimatization in the animal colony, rats were given a single i.p. injection of 1,2-dimethylhydrazine (DMH; 180 mg/kg i.p.; Sigma Chemical) in NaCl (9 g/L) to induce colon carcinogenesis. Seven days later, they were randomly allocated to one of three groups (12 rats per group) and were exposed to 0 (control), 200 μg/kg of BW/day or 10 mg/kg of BW/day of E171 in drinking water for 100 days. In a second series of experiments dedicated to the study on ACF initiation, after a similar period of acclimatization, the rats were directly exposed to 0 (control) or 10 mg/kg of BW/day of E171 in drinking water for 100 days. When the experiments were completed (i.e., promotion or initiation), the animals were killed in random order. The colons were coded, stained with methylene blue (0.1%) for 6 min, and scored for preneoplastic lesions (ACF) following Bird’s procedure^62^. The number of ACF per colon and the number of crypts per ACF were counted under a light microscope at 40X magnification in duplicate by two readers who were blinded to sample origins.

### Statistical analysis

The results are expressed as the mean ± s.e.m. Statistical significance was assessed using Prism 4 software (GraphPad). Student’s *t*-test, one-way ANOVA or the Kruskal-Wallis test, followed by *post hoc* tests were used, or contingency analysis followed by Fisher’s exact test was used, when appropriate. A *P* value of <0.05 was considered significant.

## Additional Information

**How to cite this article**: Bettini, S. *et al*. Food-grade TiO_2_ impairs intestinal and systemic immune homeostasis, initiates preneoplastic lesions and promotes aberrant crypt development in the rat colon. *Sci. Rep.* **7**, 40373; doi: 10.1038/srep40373 (2017).

**Publisher's note:** Springer Nature remains neutral with regard to jurisdictional claims in published maps and institutional affiliations.

## Supplementary Material

Supplementary Information
