# Supplementary material for: Food-grade TiO2 impairs intestinal and systemic immune homeostasis, initiates preneoplastic lesions and promotes aberrant crypt development in the rat colon
Source: Sci Rep. 2017 Jan 20;7:40373. doi: 10.1038/srep40373 (PMC5247795; doi:10.1038/srep40373)
Supplement: Supplementary Information [file srep40373-s1.pdf]

Supplementary Information

Title: Food-grade TiO_2 impairs intestinal and systemic immune homeostasis, initiates preneoplastic lesions and promotes aberrant crypt development in the rat colon

Sarah Bettini[1], Elisa Boutet-Robinet[1], Christel Cartier[1], Christine Comera[1], Eric Gaultier[1], Jacques Dupuy[1], Nathalie Naud[1], Sylviane Taché[1], Patrick Grysan[2], Solenn Reguer[3], Nathalie Thieriet[4], Matthieu Réfrégiers[3], Dominique Thiaudière[3], Jean-Pierre Cravedi[1], Marie Carrière[5,6], Jean-Nicolas Audinot[2], Fabrice H Pierre[1], Laurence Guzylack-Piriou[1,*], and Eric Houdeau[1,*]

[1]Toxalim (Research Centre in Food Toxicology), Université de Toulouse, INRA, ENVT, INP-Purpan, UPS, Toulouse, France; [2]Luxembourg Institute of Science and Technology (LIST), Materials Research and Technology (MRT), Advanced Instrumentation for Ion Nano-Analytics (IANA), L-4362 Esch-sur-Alzette, Luxembourg; [3]Synchrotron SOLEIL, F-91192 Gif-sur-Yvette, France; [4]French Agency for Food, Environmental and Occupational Health and Safety (ANSES), F-94701 Maisons-Alfort, France; [5]Université Grenoble-Alpes, INAC-LCIB, Laboratoire Lésions des Acides Nucléiques, 17 rue des Martyrs, F-38000 Grenoble, France; [6]CEA, INAC-SCIB, Laboratoire Lésions des Acides Nucléiques, 17 rue des Martyrs, F-38000 Grenoble, France

Supplementary Materials and Methods

Particle Characterization

Hydrodynamic diameter, polydispersity index and zeta potential of TiO_2 particles after dispersion protocol were measured by dynamic light scattering (ZetaSizer nano ZS; Malvern Instruments Ltd.). The size and shape of TiO_2 particles were determined by transmission electron microscopy (TEM) (JEOL 1200EX), operated at 80 kV (226 particles analyzed). Element composition was assessed by TEM and energy dispersive X-ray (JEOL JEM-2100),

operated at 200 kV. The X-ray absorption near edge structure (XANES) analysis was performed at the Synchrotron SOLEIL (Gif-sur-Yvette, France) on DiffAbs beamline, using a monochromatic x-ray beam obtained from a Si(111) double crystal monochromator. For XANES spectra, the beam size was basically 300x300 μm^2.

Comet Assay. The alkaline comet assay was used to detect strand breaks and alkali-labile sites as previously described[1]. PP and scraped colonic mucosa were collected in cold NaCl-ethylenediamine tetra-acetic acid (EDTA) (0.075 M/0.024 M, respectively; pH=7.5). Cells were isolated using a Dounce homogenizer, embedded in 0.7% low melting point agarose (Sigma-Aldrich) and laid on CometAssay® HT Slide (Trevigen). A parallel digestion with formamido pyrimidine glycosylase (FPG, gift from Serge Boiteux), allowing the detection of oxidative damages, was performed as already described[2]. Fifty cells per slide and 2 slides per sample were analyzed. The extent of DNA damage was evaluated for each cell through the measurement of intensity of all tail pixels divided by the total intensity of all pixels in head and tail of comet. The median from these 100 values was calculated, and named % tail DNA.

Myeloperoxidase Activity Assay. For assessment of neutrophilia in intestinal mucosa, the activity of the enzyme myeloperoxidase (MPO), a marker of polymorphonuclear neutophils granules, was determined in tissue segments of the jejunum and colon (1 cm) as previously described[3].

***Ex Vivo* Cytotoxicity and Proliferation Assay**. Isolated cells from PP were labeled with CellTrace® Violet (Life Technologies) to assess proliferation. Cells were seeded on 24-well plates at $6*10^5$ cells/well in Cerrotini medium, and incubated with 2 µg/mL concanavalin A for 4 days with E171 or NM-105 (0, 37, 75 or 150 µg/mL). After washing, T cells were

stained with anti-TCRαβ (R73) and with To-pro-3 (Life Technologies) to follow cell viability by flow cytometry.

Western blot analysis for caspase-1. Colons of rats treated with vehicle or E171 (n=4 per group) for 100 days were collected in dry ice and stored at -80°C until processing. Proteins were extracted with RIPA buffer containing protease inhibitor cocktail (Roche Diagnostics), and total protein concentrations were assessed using a BCA Protein Assay kit (Thermo Scientific, Rockford, USA). Equal amounts of protein extracts (30 μg) were separated in 12% FastCast TGX Bio-Rad gels and transferred onto a blotting membrane. The membrane was blocked for 1 h at RT with Odyssey blocking buffer (Rockland, Tebu-bio, France) and then incubated overnight at 4°C with primary mouse anti-caspase-1 antibody (R&D Systems; 1:1000 in blocking buffer). After washing, the membranes were incubated for 1 h at RT with secondary fluorescent IRDye *680 anti-mouse antibody (LI-COR, Lincoln, USA; 1:10000 in blocking buffer). An Odyssey Infrared Imaging Scanner (LiCor ScienceTec, Les Ulis, France) was used to detect the intensity of full length (45 kD) and cleaved (20 kD) caspase-1 protein bands.

Supplementary Results

Size and Crystal Form of Titanium Dioxide. The referent NM-105 batch of P25 has been previously characterized[4], and displayed mixed crystallinity with anatase the predominant form (85% anatase : 15% rutile), and a mean particle diameter of 22±1 nm (range 10-45 nm). As shown in Figure S1a, TEM-EDX analysis showed the E171 sample (i.e. food-grade TiO_2) close to purity. Primary particle size distribution ranged from 20 to 340 nm (118±53 nm), and 44.7% were below 100 nm in diameter (Fig. S1b). Furthermore, the hydrodynamic diameter of E171 in our study was of 373±20 nm (Supplementary Fig. S1c), consistent with

measurements reported for other commercial food-grade TiO_2 ranging from 127 to 504 nm depending on the supplier[5-6]. The zeta potentials were 5.03±0.02 mV and -23.9±2.4 mV for NM-105 and E171, respectively (Supplementary Fig. S1c), suggesting a higher stability of the E171 suspension in water as compared to NM-105. In Figure S1d, the fact that the XANES spectra of titanium (Ti) overlapped between E171 and NM-105 indicated similar crystal form, thus primarily composed of TiO_2 anatase as commonly reported for other food-grade TiO_2[6-7]. Altogether, these findings made our commercial E171 batch a representative TiO_2 food additive for oral toxicity study.

Supplementary Figures

a

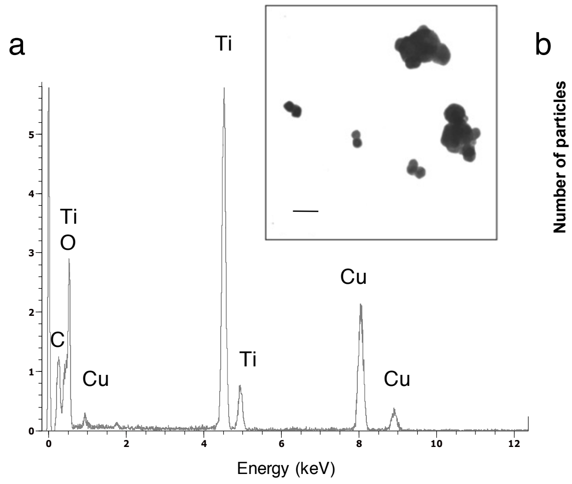

b

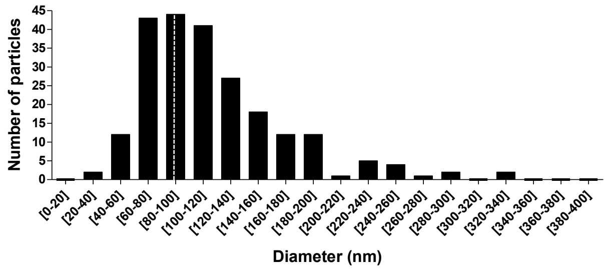

c

	Z average	PdI	(mV)
NM-105	192.23 ê 5.43	0.133 ê 0.014	5.013 ê 0.025
E171	373.5 ê 20.22	0.316ê0.048	-23.9 ê 2.36

d

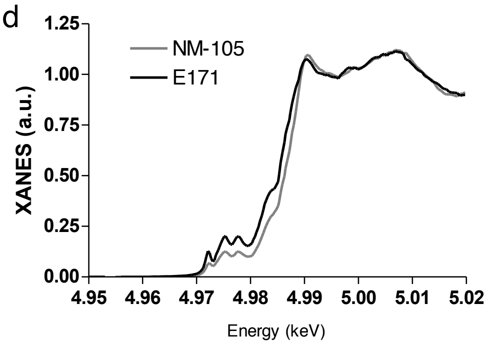

Figure S1. Physico-chemical characteristics of E171 and NM-105 TiO_2 particles. (**a**) EDX spectrum and TEM image recorded on E171. EDX data indicates TiO_2 particles to be close to purity, being devoid of surface coatings with alumina, phosphate or silica (the presence of copper corresponded to the TEM-EDX grid used as a sample support). TEM image shows E171 particles displaying a spherical shape, found isolated and in small agglomerates when dispersed in water before oral administration in rats (scale bar 200 nm). (**b**) Primary diameter distribution of E171 particles, measured from 4 independent TEM images and a total of 226 particles. Dashed line shows 100 nm. (**c**) Hydrodynamic diameter (Z average), polydispersity index (PdI) from DLS measurements and zeta potential (ζ) of E171 water suspension after dispersion protocol according to Nanogenotox standard operating procedure. (**d**) XANES spectra of E171 and NM-105 materials measured with synchrotron x-ray beam on the raw powders. The fact that pre-peaks and post-edge XANES features overlay between TiO_2 materials indicates similar crystal form, i.e., mainly composed of anatase.

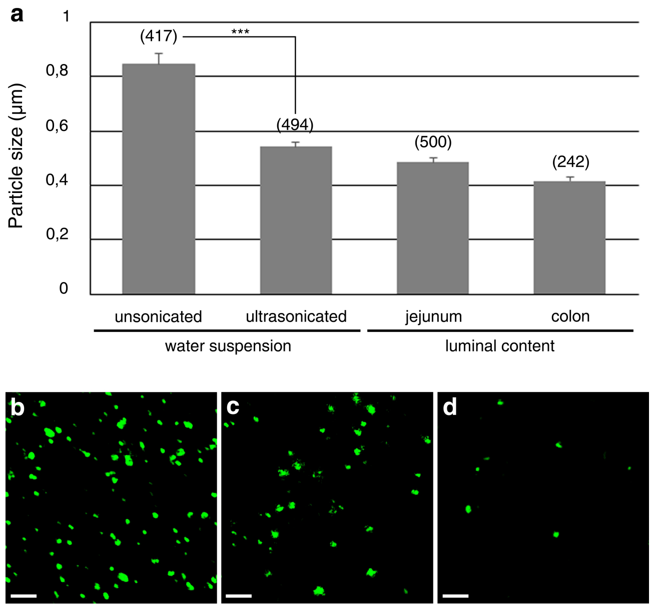

Figure S2. TiO_2 particle size determination using confocal microscopy in water suspension (before and immediately after ultrasonication) and in the luminal contents of the jejunum and the colon collected 4 hours after a single gavage of 10 mg/kg E171 (n=4 rats). The laser reflection of TiO_2 was detected with a magnification given 1 pixel = 50 nm. (a) Particle size measurements using Image J and Student's *t*-test (***P<0.001) for statistical analysis. Note the number of counted particles is indicated in brackets. **(b, c, d)** Representative confocal micrographs of ultrasonicated TiO_2 particles in water suspension before the gavage **(b)**, and along the intestine in the luminal content of the jejunum **(c)** and the colon **(d).**

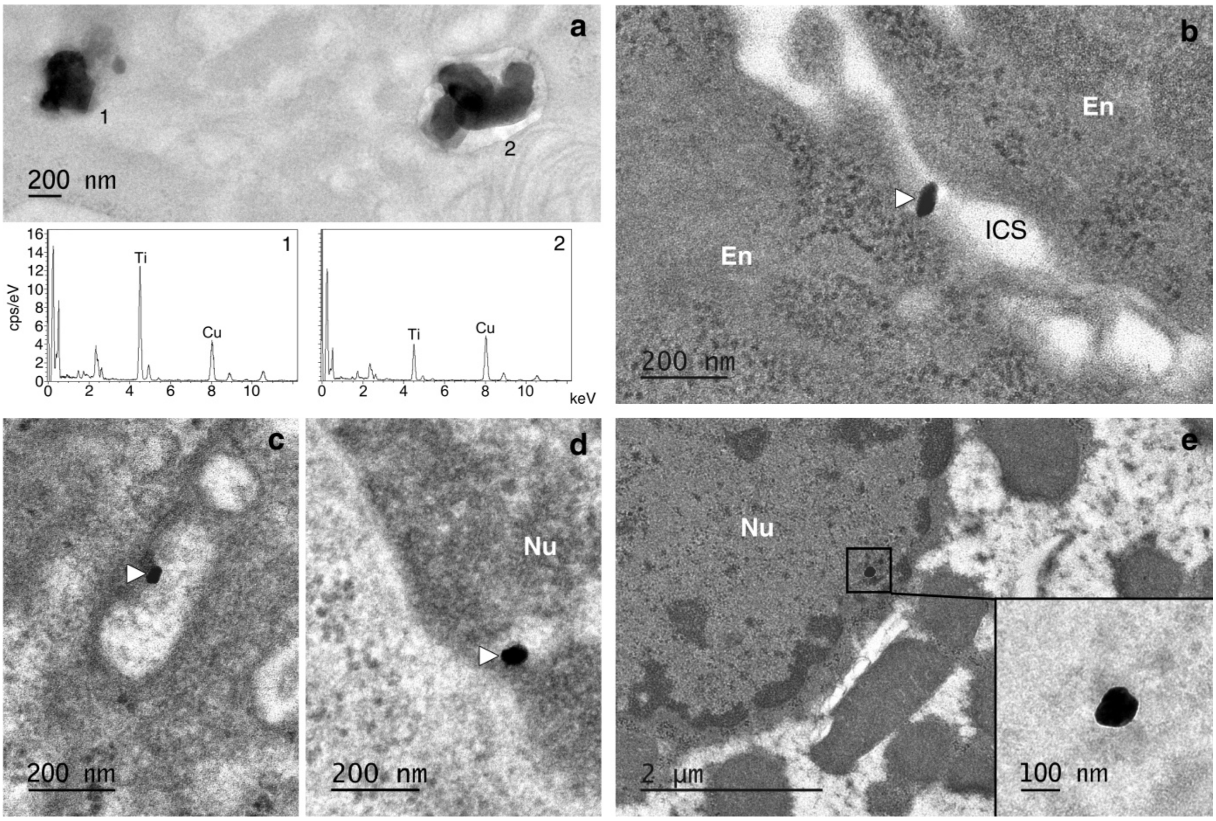

Figure S3. Transmission electron micrographs showing tissue distribution of TiO_2 particles in the rat intestine and the liver after 7 days of oral treatment with E171.

(**a**) TEM micrograph and corresponding TEM-EDX spectra of E171 TiO_2-particles in the colonic mucosa. (**b**) Colonic epithelium section showing E171 nanoparticle (white arrowhead) in the intercellular space (ICS) between enterocytes (En). (**c-d**) Peyer's patch section (jejunum) showing E171 nanoparticle (white arrowhead) in the cytoplasm (**c**) and in the nucleus (Nu) of an immune cell (**d**). (**e**) Liver section showing E171 nanoparticle in the nucleus of one hepatocyte.

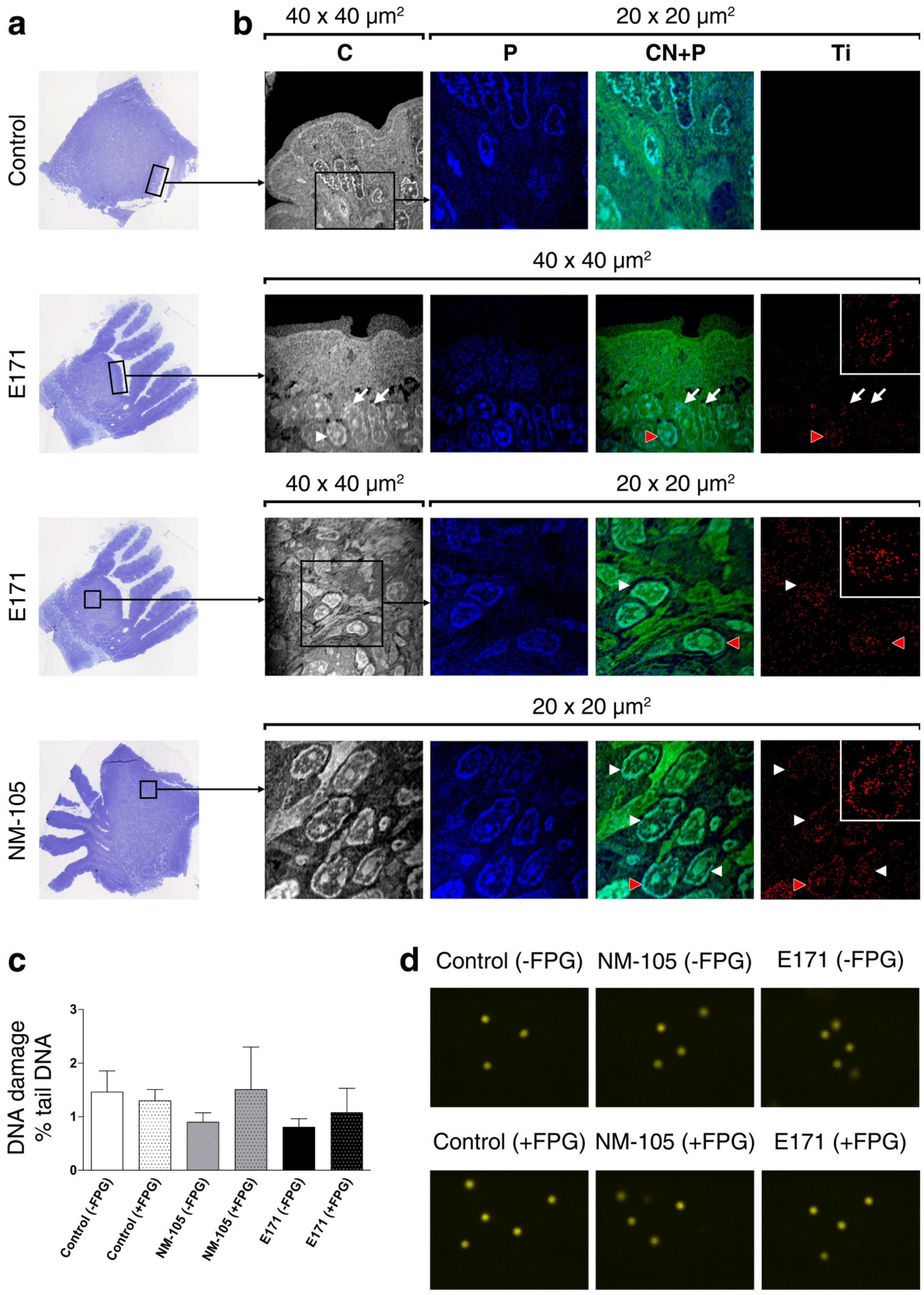

Figure S4. NanoSIMS analyses of Ti distribution in PP and genotoxicity assessment after 7 days of oral treatment with NM-105 or E171. (**a**) TEM images at low magnification

on ultra-thin PP sections, and (**b**) nanoSIMS images of corresponding fields for elemental distribution of carbon-nitrogen $^{12}C^{14}N$ (black and white), phosphorus ^{31}P (blue), merge of ^{31}P and $^{12}C^{14}N$ (blue/green), and titanium oxide clusters $^{48}Ti^{16}O$ (red). Raster sizes in (**b**) were 40x40 or 20x20 μm^2. The two first horizontal series of micrographs show PP tissue sections from the surface epithelium in control rats and from rats exposed to E171 for 7 days. The two following series show the central zone of PP tissue sections after E171 and NM-105 treatment for 7 days. Note that the nucleus of immune cells (white arrowheads) is enriched in Ti elements in contrast to epithelial cell nuclei (white arrows). Red arrowheads indicate enlarged fields in upper squares. In (**c**), note the absence of genotoxic effects on PP cells as compared to controls evaluated with alkaline comet assay without or with FPG (revealing DNA strand breaks and oxidized DNA bases). (**d**) Representative images of (**c**). Data are means±s.e.m, and no statistical difference between groups was observed (Kruskall-Wallis test, n=5 rats/group).

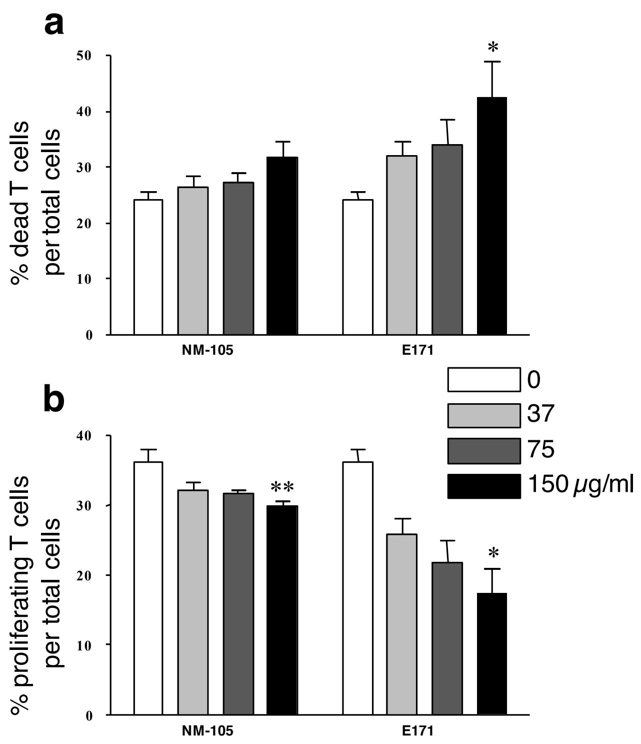

Figure S5. Dose-effect of TiO_2 on T cell viability and proliferation *in vitro*. Cell suspensions from PP harvested from untreated rats were exposed for 4 days to concanavalin-A (a T-cell mitogen) in the presence or absence of various concentrations of NM-105 or E171. (**a**) Frequency of dead T cells was evaluated by flow cytometry analysis of To-pro-3 staining. (**b**) Frequency of proliferative T cells was determined by measuring cell division with CellTrace® Violet labeling and flow cytometry analysis. All data are means±s.e.m from 3 to 4 independent experiments. *P<0.05, **P<0.01 *vs.* control by Kruskall-Wallis test followed by Dunn's multiple comparison test.

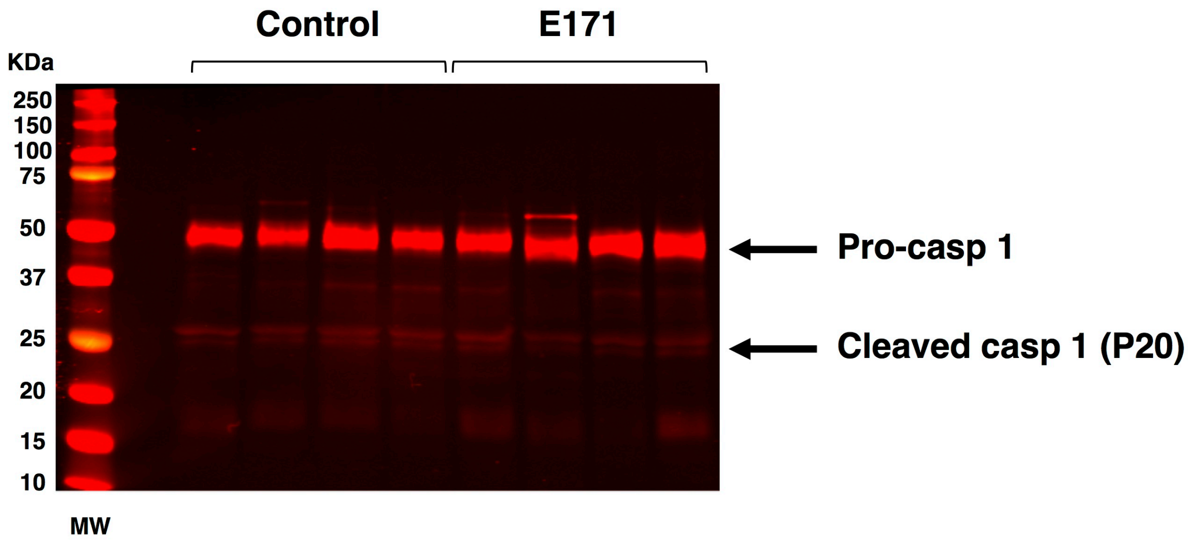

Figure S6. Western blot analysis of caspase-1 activation in the rat colonic mucosa after 100 days of oral treatment with E171. No detectable increase of caspase-1 cleavage was observed in the colonic mucosa of E171-exposed rats.

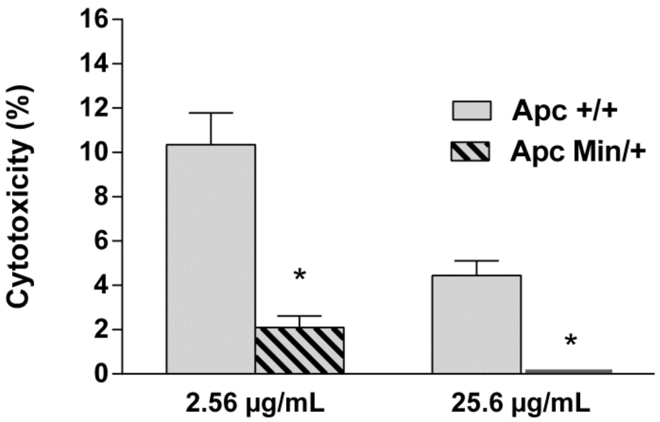

Figure S7. Exposure to NM-105 TiO_2 induces preferential cytotoxicity against preneoplastic cells. Normal $Apc^{+/+}$ and preneoplastic $Apc^{Min/+}$ colon cells were exposed 24h to 2.56 µg/mL or 25.6 µg/mL TiO_2. *P<0.05 *vs.* Apc +/+, Student's *t*-test.

	Jejunum			Colon		
	Control	NM-105	E171	Control	NM-105	E171
MPO	3160±424	2424±355^{ns}	2862±526^{ns}	686±119	611±146^{ns}	609±130^{ns}
TNF-α	nd	nd	nd	5.4±1	8.3±0.3^{ns}	5.8±1.2
IL-10	nd	nd	nd	18±2.6	19±2.6	14±2.3
IL-1β	225±55	284±100^{ns}	145±46^{ns}	189±17	175±31	178±16
IFN-γ	nd	nd	nd	nd	nd	nd
IL-17	nd	nd	nd	9.9±1.1	10.2±1.9	10±0.3

Table S1. A 7 days oral treatment with E171 or NM-105 did not provoke inflammation in the jejunal and colonic mucosa. Cytokine concentrations (pg/mg of proteins) and myeloperoxydase activity (MPO, U/mg of protein) were evaluated in jejunal and colonic tissues by ELISA and enzyme activity assay, respectively. Data are means±s.e.m. (n=5 rats/group); ns: not significant *vs.* control by Kruskall-Wallis test followed by Dunn's multiple comparison test; nd: not detectable.

References

1 Lebailly, P. *et al.* DNA damage in B and T lymphocytes of farmers during one pesticide spraying season. *Int Arch Occup Environ Health* **88**, 963-972 (2015).

2 Azqueta, A., Arbillaga, L., Lopez de Cerain, A. & Collins, A. Enhancing the sensitivity of the comet assay as a genotoxicity test, by combining it with bacterial repair enzyme FPG. *Mutagenesis* **28**, 271-277 (2013).

3 Menard, S. *et al.* Food intolerance at adulthood after perinatal exposure to the endocrine disruptor bisphenol A. *FASEB J* **28**, 4893-4900 (2014).

4 Rasmussen, K. *et al.* Titanium Dioxide, NM-100, NM-101, NM-102, NM-103, NM-104, NM-105: Characterisation and Physico-Chemical Properties. Report No. EUR 26637 EN, (JRC, 2014).

5 Weir, A., Westerhoff, P., Fabricius, L., Hristovski, K. & von Goetz, N. Titanium Dioxide Nanoparticles in Food and Personal Care Products. *Environ Sci Technol* **46**, 2242-2250 (2012).

6 Yang, Y. *et al.* Characterization of food-grade titanium dioxide: the presence of nanosized particles. *Environ Sci Technol* **48**, 6391-6400 (2014).

7 Faust, J. J., Doudrick, K., Yang, Y., Westerhoff, P. & Capco, D. G. Food grade titanium dioxide disrupts intestinal brush border microvilli in vitro independent of sedimentation. *Cell Biol Toxicol* **30**, 169-188 (2014).
